# Corneal esthesiometry between 2000 and 2024: A bibliometric and knowledge mapping analysis

**DOI:** 10.1097/MD.0000000000042383

**Published:** 2025-05-09

**Authors:** Javier Lozano-Sanroma, Alberto Barros, Juan Queiruga-Piñeiro, Ignacio Alcalde, Rosa Alvarado-Villacorta, Luis Fernández-Vega Cueto-Felgueroso, Jesús Merayo-Lloves

**Affiliations:** a Optometry Department, University Institute Fernández-Vega, Ophthalmological Research Foundation, Oviedo, Spain; b Ocular Surface and Nerve Regeneration Research, University Institute Fernández-Vega, Ophthalmological Research Foundation, Oviedo, Spain; c Institute for Health Research of the Principality of Asturias (ISPA), University of Oviedo, Oviedo, Spain; d Research Department, University Institute Fernández-Vega, Ophthalmological Research Foundation, Oviedo, Spain; e Cornea and Lens Unit, University Institute Fernández-Vega, Ophthalmological Research Foundation, Oviedo, Spain; f Director of Research, University Institute Fernández-Vega, Ophthalmological Research Foundation, Oviedo, Spain.

**Keywords:** bibliometric analysis, corneal esthesiometry, knowledge mapping, publications metrics

## Abstract

**Background:**

Corneal esthesiometry plays a key role in assessing the integrity of the ocular surface. Its importance lies in the fact that several eye and systemic conditions can alter the corneal sensitivity. This is evidenced by the emergence of new devices to measure this parameter in recent years.

**Methods:**

Publications found in Web of Science and Scopus databases from 2000 to 2024 were analyzed. Microsoft Excel, Rayyan, and VOSviewer software were used.

**Results:**

A total of 556 articles were included in the study. Andrew JM Boulton had the greatest impact, with the highest *h*-index. The Centre for Contact Lens Research, School of Optometry, University of Waterloo (Canada), was the most prolific institution, with 25 articles published. The United States led the ranking of countries, with 81 publications. Six keyword clusters were identified, encompassing neurophysiology, dry eye, ocular pathology, diabetic neuropathy, structural nerve assessment, and refractive surgery.

**Conclusion:**

This study reports on who, why, how many, and where corneal esthesiometry has been studied, through a bibliometric analysis. The studies centered on sensory physiology, ocular surface disease, and dry eye disease. These emerging trends highlight new clinical, diagnostic, and research perspectives, which may guide future investigations and contribute to more precise strategies for the treatment of ocular surface conditions.

## 1. Introduction

**Figure 1.**
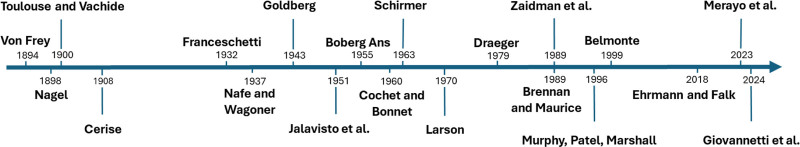
Timeline of the use of different esthesiometers has developed throughout history.

Corneal esthesiometry is the measurement of the cornea’s sensitivity to a stimulus, which can be tactile, pressure-based, chemical, or thermal. To measure sensitivity levels, instruments known as esthesiometers were developed, with the earliest models dating back to 1894, using horsehairs of varying lengths. Subsequently models have enabled the assessment of corneal sensitivity to chemical and thermal stimuli (see Fig. 1).^[1–7]^ The interest in determining corneal sensitivity is well justified, as various ocular and systemic conditions, as well as therapeutic and surgical treatments, can lead to alterations in corneal sensitivity.^[8]^

Bibliometrics is a branch of scientometrics that uses mathematical and statistical methods to analyze scientific production and its impact. It is used through indicators that measure the activity and relationships between scientific publications. These indicators evaluate the number of publications in a field, or the citations obtained by an article. Relationship indicators are also studied by analyzing the connections between publications, authors, or keywords.^[9]^ The origin of bibliometric analysis dates to 1963, although the concept was formulated as early as 1956^[10]^ and even in the early 1900s.^[11]^ Specifically, in the field of ophthalmology, it began in 2001.^[12]^ Knowledge mapping domain analysis provides a new method for literature mining and revealing the core structure of scientific knowledge.^[13]^

In recent years, new devices have been developed to improve upon the Cochet-Bonnet esthesiometry, which is considered the gold standard. This motivated us to conduct the present bibliometric study, which, to our knowledge, has not been done before, to learn about relevant aspects that have been published and trends in corneal esthesiometry.

## 2. Methods

### 2.1. Search criteria

**Figure 2.**
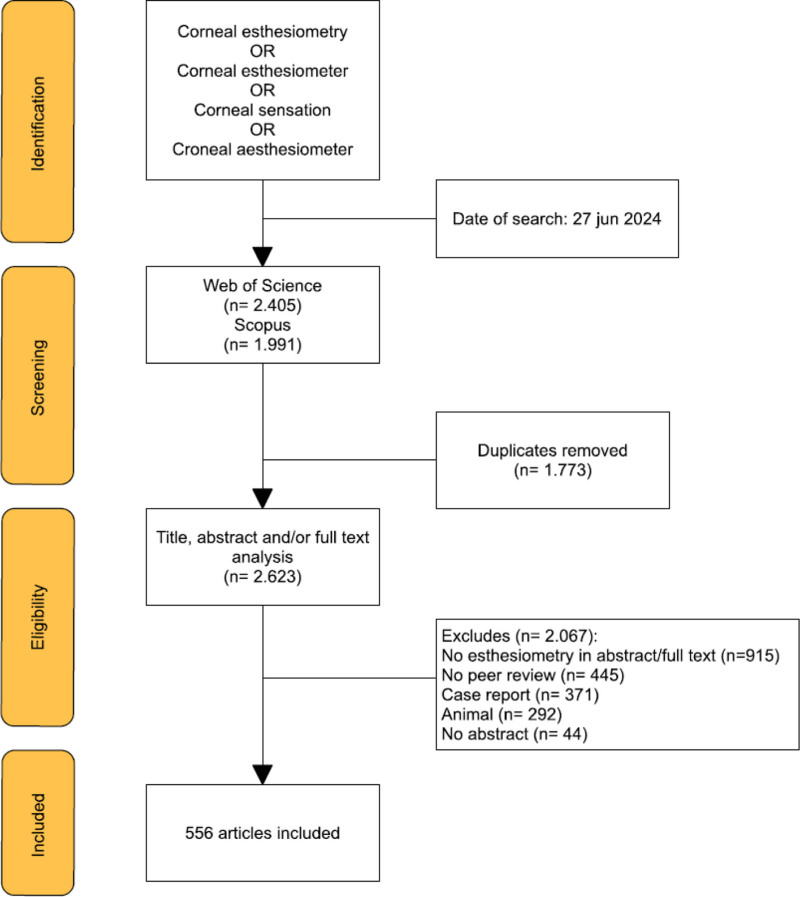
Flow chart for article selection.

The web of science (WoS) and Scopus databases were searched until June 27,2024 using the terms “Corneal esthesiometry,” “Corneal esthesiometer,” “Corneal sensation” and, “Corneal aesthesiometer,” with the Boolean operator “OR” (see Fig. 2). Peer-reviewed articles published between 2000 and 2024 were included. Conference or meeting abstracts and case reports were discarded. No language or regional limits were set. Only human studies were included.

### 2.2. Analysis tools

The independent selection of articles was carried out by 3 blinded researches using the Rayyan software tool.^[14]^ The selected articles were exported and analyzed with Microsoft Excel 365 software (Microsoft Corporation, 2024). To study the co-occurrence of authors and keywords and construct the knowledge map, VOSviewer software was used.^[15]^

### 2.3. Data analysis

The number of articles published per year; the authors, journals, institutions, and countries with the highest publication output; and the most cited articles were analyzed. It was necessary to merge some author names and institutional affiliations due to inconsistences in how they were recorded, which resulted in duplicate entries. The co-occurrence networks of the most relevant keywords and authors were also examined. Additionally, the *h*-indexes of the authors with the highest number of publications were calculated.

This study followed the BIBLIO guide^[16]^ for its elaboration as recommended by the EQUATOR Reporting Guidelines.

### 2.4. Ethical statement

The data for this study were obtained from peer-reviewed articles published in indexed journals and found in public databases. In addition, it did not involve human or animal subjects and therefore did not require ethical approval.

## 3. Results

**Figure 3.**
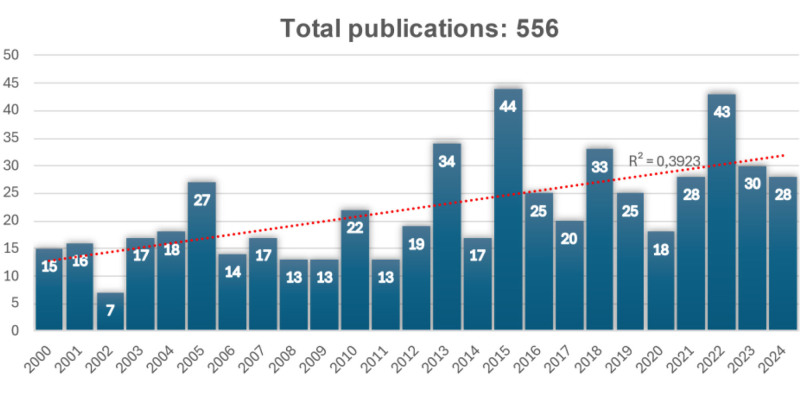
Number of published articles on esthesiometry from 2000 to mid-2024.

A total of 556 articles were analyzed. The number of publications per year was inconsistent, with the most articles published in 2015 (n = 44), followed by 2022 (n = 43), and 2013 (n = 34). Up to the first 6 months of 2024, a total of 28 articles had been published (see Fig. 3). Among the 20 most cited articles, the ones with the lowest number of citations had 144 (Scopus) and 147 (WoS) citations, respectively, and the highest number of citations was for *“Corneal structure and sensitivity in type 1 diabetes mellitus,”* with 321 citations in Scopus and 355 in WoS.

### 3.1. Authors and h-index

**Figure 4.**
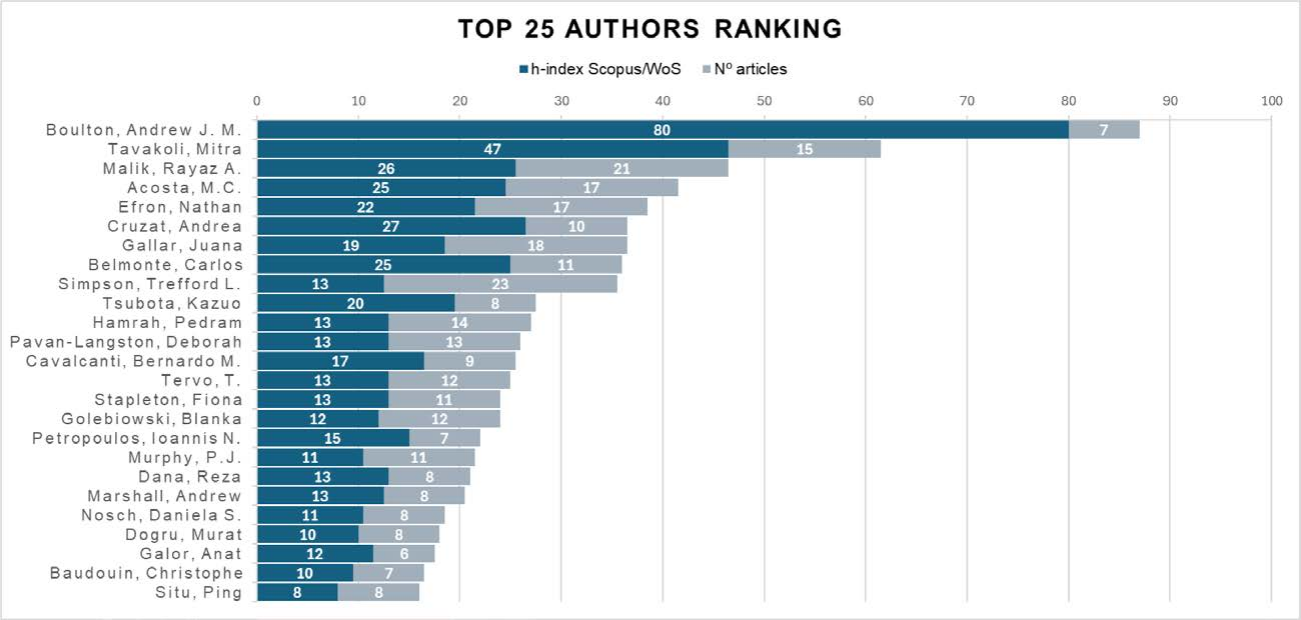
The 25 authors with the highest *h*-indexes.

A total of 2217 authors were identified. Figure 4 shows the 25 authors with the highest *h*-indexes and numbers of articles.

### 3.2. Journals and articles

The 20 journals that published the most articles were listed as ophthalmology specialties in the WoS (see Table 1), except for Plos One – multidisciplinary sciences – and the Journal of Refractive Surgery – surgery – as well as the British Journal of Ophthalmology – sensory systems – in Scopus.

**Table 1:** The 20 journals with the highest number of publications on esthesiometry, their ranking in Web of Science and Scopus in 2023 and their publisher.

		Web of Science			Scopus			
		JIF			IF CiteScore	*Q* percentile	Rank†	
Journal	Items	2023	Percentile	Rank*				Publisher
Cornea	54	1.9	54.2	44/95	5.2	82	25/137	Wolters Kluwer Health
Investigative Ophthalmology and Vision Science	42	5	94.2	6/95	6.9	88	16/137	Association for Research in Vision and Ophthalmology Inc.
American Journal of Ophthalmology	25	4.1	90	10/95	9.2	94	8/137	Elsevier
Ophthalmology	22	13.1	98.4	2/95	22.3	98	2/137	
Journal of Cataract and Refractive Surgery	19	2.6	73.2	26/95	5.6	88	23/137 to 63/551	Wolters Kluwer Health
The Ocular Surface	17	5.9	96.3	4/95	11.6	97	4/137	Elsevier
Journal of Refractive Surgery	17	2.9	78.4	21/95	5.1	85	78/551	Slack, Inc.
British Journal of Ophthalmology	16	3.7	86.8	13/95	10.3	96	2/42	BMJ Publishing Group
Eye	16	2.8	76.3	23/95	6.4	86	19/137	Springer Nature
Graefes Archive for Clinical and Experimental Ophthalmology	16	2.4	67.9	31/95	5.4	82	24/137	
Optometry and Vision Science	14	1.6	41.6	56/95	2.8	55	61/137	Wolters Kluwer Health
Journal of The Korean Ophthalmology Society	14	0.1	2.6	93/95	0.2	9	125/137	Korean Ophthalmological Society
Current Eye Research	13	1.7	45.8	52/95	4.6	77	32/137	Taylor and Francis
Plos One	13	2.9	77.2	31/134	6.2	89	18/171	Public Library of Science
Clinical and Experimental Optometry	11	1.7	45.8	52/95	4.1	71	40/137	Tailor and Francis
Experimental Eye Research	8	3	81.6	18/95	6.8	87	18/137	Elsevier
Eye and Contact Lens-Science and Clinical Practice	8	2	60.5	38/95	4.5	76	33/137	Wolters Kluwer Health
European Journal of Ophthalmology	8	1.4	36.3	61/95	3.6	66	47/137	Sage Publications Inc.
Ophthalmic and Physiological Optics	7	2.8	76.3	23/95	5.1	79	28/137	John Wiley and Sons
International Journal of Ophthalmology	7	1.9	54.2	44/95	2.5	52	65/137	Press of International Journal of Ophthalmology

IF = impact factor, JIF = journal impact factor.

* The WoS database contains 95 journals in the ophthalmology category, 134 in the multidisciplinary sciences category and 290 in the surgery category.

† The Scopus database contains 137 journals in the ophthalmology category, 551 in the surgery category, 171 in the multidisciplinary sciences category and 42 in the sensory systems category.

In Table 2, the 20 most referenced articles can be found, ranging from more than 300 citations for the article *“Corneal structure and sensitivity in type 1 diabetes* mellitus,” to more than 140 for *“The Relationship between Subbasal Nerve Morphology and Corneal Sensation in Ocular Surface Disease.”*

**Table 2:** Ranking of the 20 most cited articles related to esthesiometry between 2000 and mid-2024.

Title	Yr	Citations		Authors	Journal
		WoS	Scopus		
Corneal structure and sensitivity in type 1 diabetes mellitus	2000	355	321	Rosenberg ME, Tervo T, Immonen IJ, et al	IOVS
Relation between corneal innervation with confocal microscopy and corneal sensitivity with noncontact esthesiometry in patients with dry eye	2007	275	277	Benitez-del-Castillo JM, Acosta MC, Wassfi Mohamed A, et al	
Corneal Confocal Microscopy: A novel noninvasive test to diagnose and stratify the severity of human diabetic neuropathy	2010	266	293	Tavakoli Mitra, Quattrini C, Abbott C, et al	Diabetes Care
Corneal Sensation and Subbasal Nerve Alterations in Patients with Herpes Simplex Keratitis: An In Vivo Confocal Microscopy Study	2010	227	254	Hamrah Pedram, Cruzat Andrea, Dastjerdi MH, et al	Ophthalmology
Tear function and ocular surface changes in noninsulin-dependent diabetes mellitus	2001	226	273	Dogru Murat, Katakami C, Inoue M.	
Femtosecond laser vs mechanical keratome flaps in wavefront-guided laser in situ keratomileusis – Prospective contralateral eye study	2005	224	230	Durrie DS, Kezirian GA	JCRS
Performance of Tear Osmolarity Compared to Previous Diagnostic Tests for Dry Eye Diseases	2010	213	220	Versura P, Profazio V, Campos EC	Current Eye Research
Recovery of corneal subbasal nerve density after PRK and LASIK	2005	212	205	Erie JC, McLaren JW, Hodge DO, et al	AJO
Corneal confocal microscopy detects early nerve regeneration after pancreas transplantation in patients with type 1 diabetes	2007	198	201	Mehra S, Tavakoli Mitra, Kallinikos PA, et al	Diabetes Care
Effect of myopic LASIK on corneal sensitivity and morphology of subbasal nerves	2000	196	225	Linna TU, Vesaluoma MH, Pérez-Santonja JJ, et al	IOVS
Dry eye after laser in situ keratomileusis	2001	190	227	Toda I, Asano-Kato N, Komai-Hori Y, et al	AJO
Dry Eye Disease after Refractive Surgery Comparative Outcomes of Small Incision Lenticule Extraction vs LASIK	2015	183	220	Denoyer A, Landman E, Trinh L, et al	Ophthalmology
Decreased corneal sensitivity in patients with dry eye	2005	171	190	Bourcier T, Acosta MC, Borderie V, et al	IOVS
Confocal microscopy in vivo in corneas of long-term contact lens wearers	2002	171	172	Patel SV, McLaren JW, Hodge DO, et al	
Corneal reinnervation after LASIK: Prospective 3-year longitudinal study	2004	169	178	Calvillo MP, McLaren JW, Hodge DO, et al	
The incidence and risk factors for developing dry eye after myopic LASIK	2006	168	194	De Paiva CS, Chen Z, Koch DD, et al	AJO
The effect of hinge position on corneal sensation and dry eye after LASIK	2003	162	167	Donnenfeld ED, Solomon K, Perry HD, et al	Ophthalmology
Corneal nerve structure and function in patients with non-Sjögren dry eye: Clinical correlations	2013	161	169	Labbé A, Liang Q, Wang Z, Zhang Y, et al	IOVS
Alterations in corneal sensitivity and nerve morphology in patients with primary Sjögren’s syndrome	2008	158	162	Tuisku IS, Konttinen YT, Konttinen LM, et al	Experimental Eye Research
The Relationship between Subbasal Nerve Morphology and Corneal Sensation in Ocular Surface Disease	2012	147	144	Labbe A, Alalwani H, Van Went C, et al	IOVS

AJO = American Journal of Ophthalmology, IOVS = Investigative Ophthalmology and Visual Science, JCRS = Journal of Cataract and Refractive Surgery.

### 3.3. Institutions

There were 7 institutions that published more than 10 papers. These were the Centre for Contact Lens Research, School of Optometry, University of Waterloo, Canada (n = 25 papers); the University of Manchester, UK (n = 22); Queensland University of Technology, Australia (n = 19); the School of Optometry and Vision Science, University of New South Wales, Australia (n = 16); the Institute of Neurosciences, Universidad Miguel Hernandez, Spain (n = 14); the Department of Ophthalmology, Keio University, Japan (n = 12); and Harvard Medical School, USA (n = 11).

### 3.4. Countries

**Figure 5.**
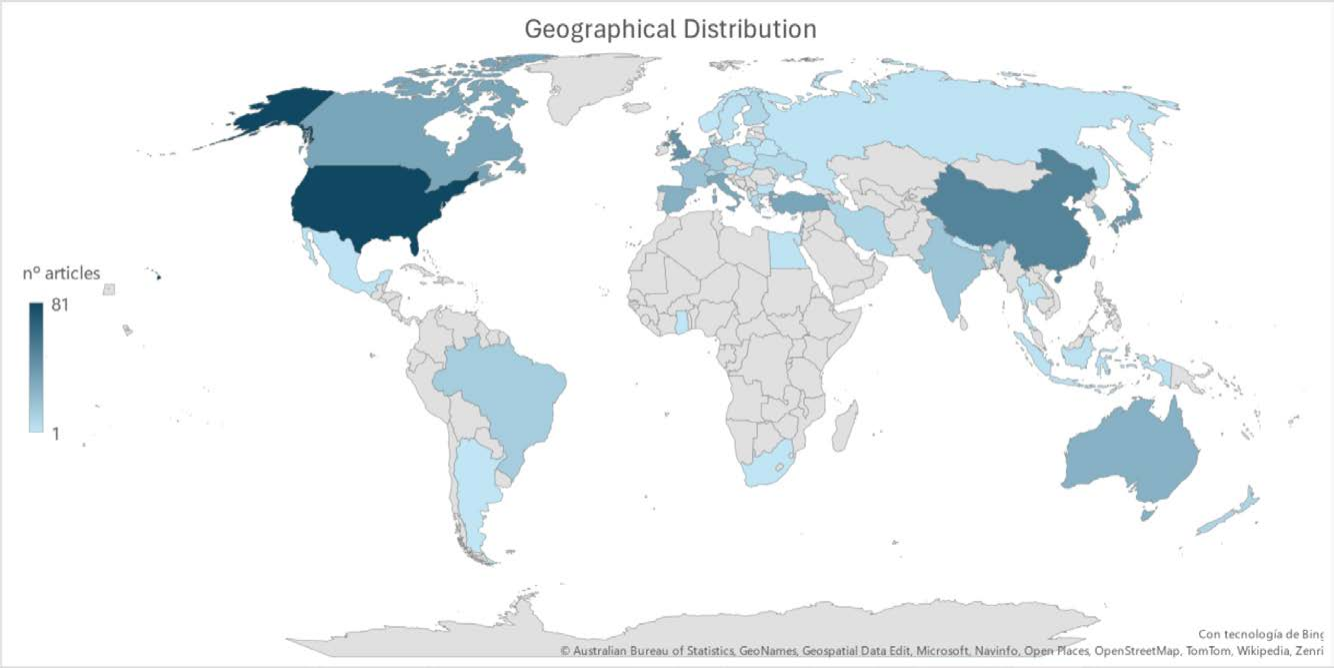
Geographical distribution of published articles on esthesiometry from 2000 to mid-2024.

A total of 44 countries published at least 1 article (see Fig. 5). Ten countries published more than 20 articles. The United States led the ranking with 81 articles, followed by China (n = 50), the United Kingdom (n = 43), Japan (n = 39), Canada (n = 33), Turkey (n = 33), Italy (n = 32), South Korea (n = 29), Spain (n = 28), and Australia (n = 27).

### 3.5. Knowledge mapping domain

**Figure 6.**
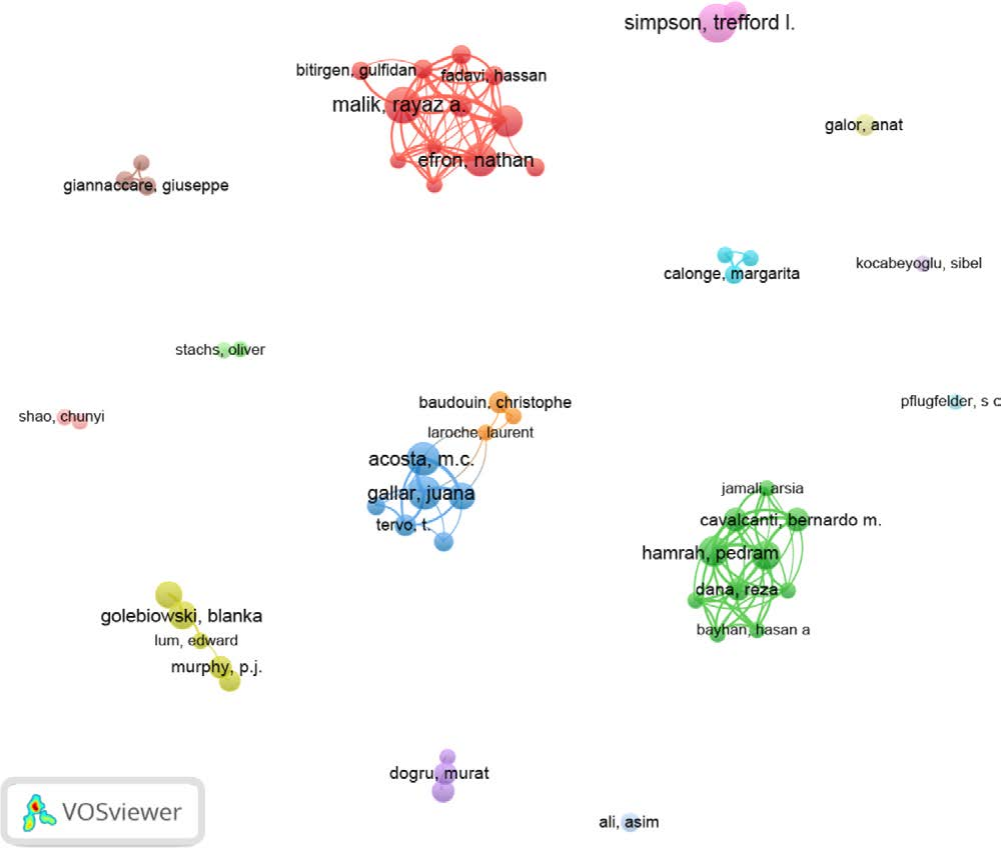
Knowledge mapping network visualization. Author co-occurrence map.

**Figure 7.**
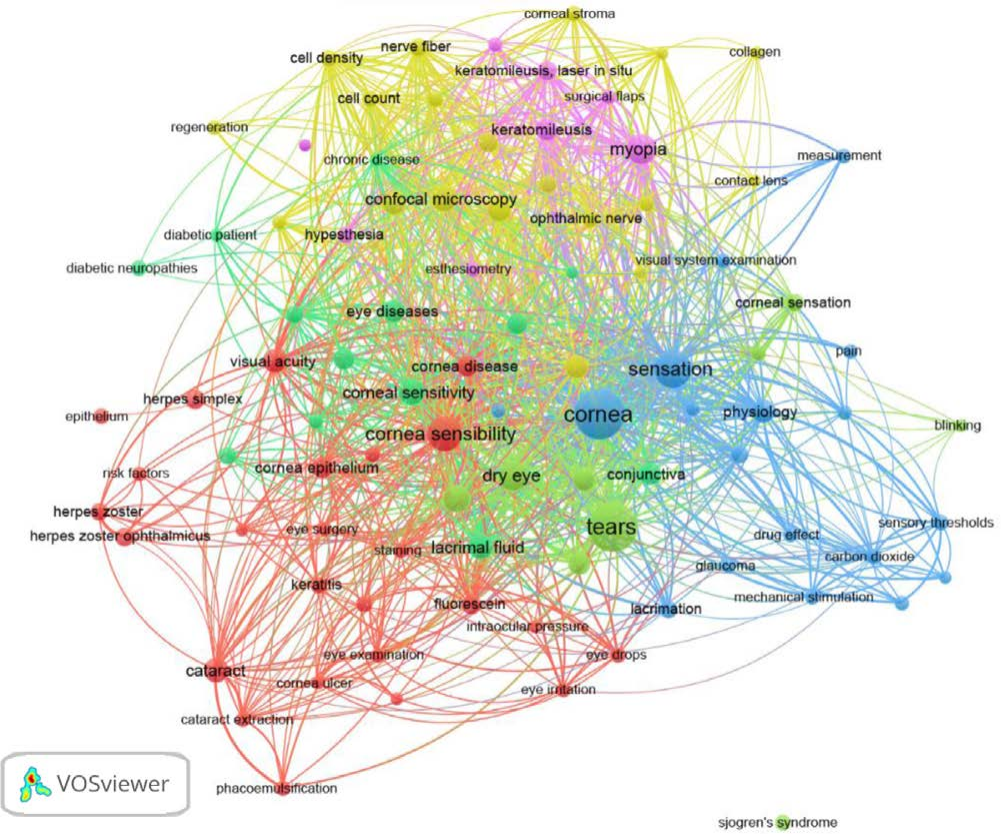
Knowledge mapping network visualization. Keyword co-occurrence map.

**Figure 8.**
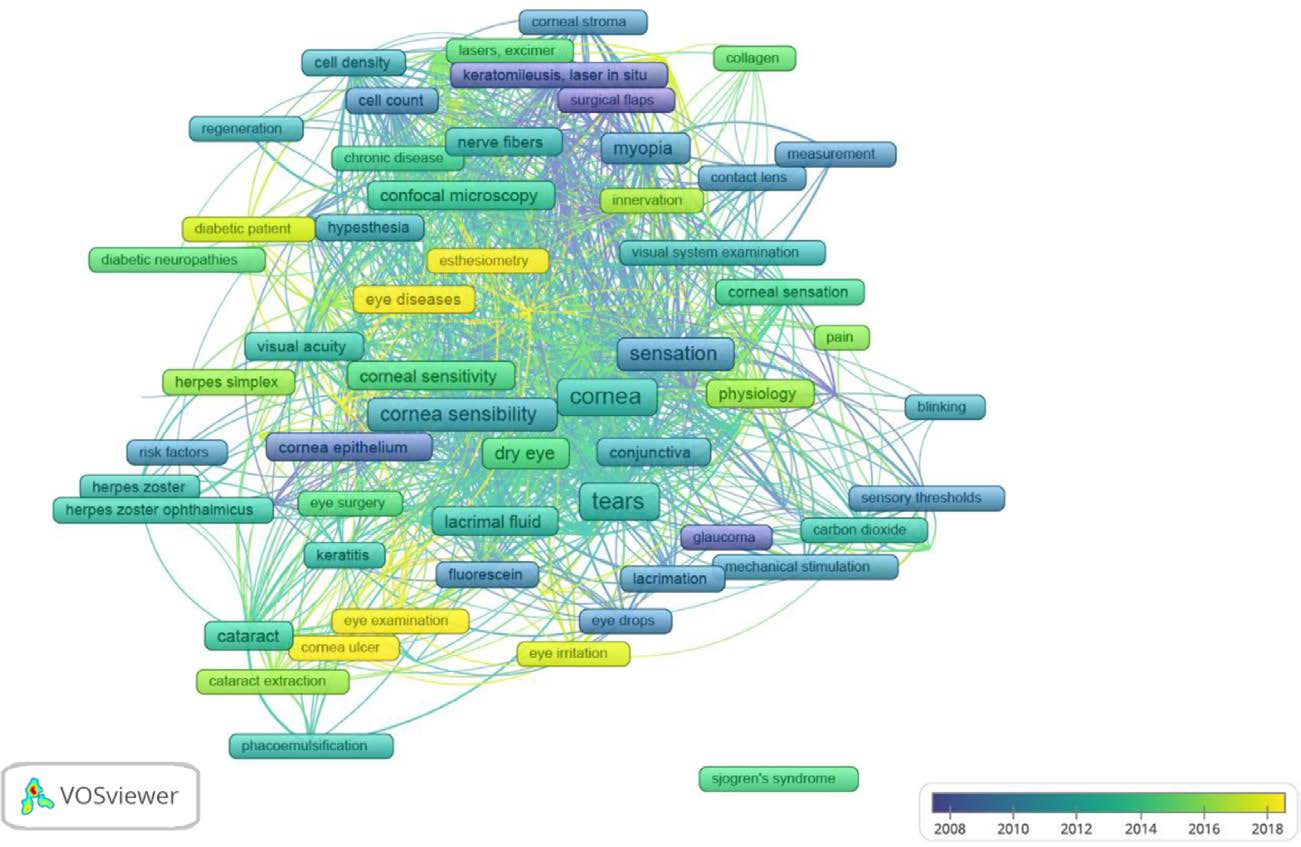
Knowledge mapping network visualization. Overlay visualization map.

**Figure 9.**
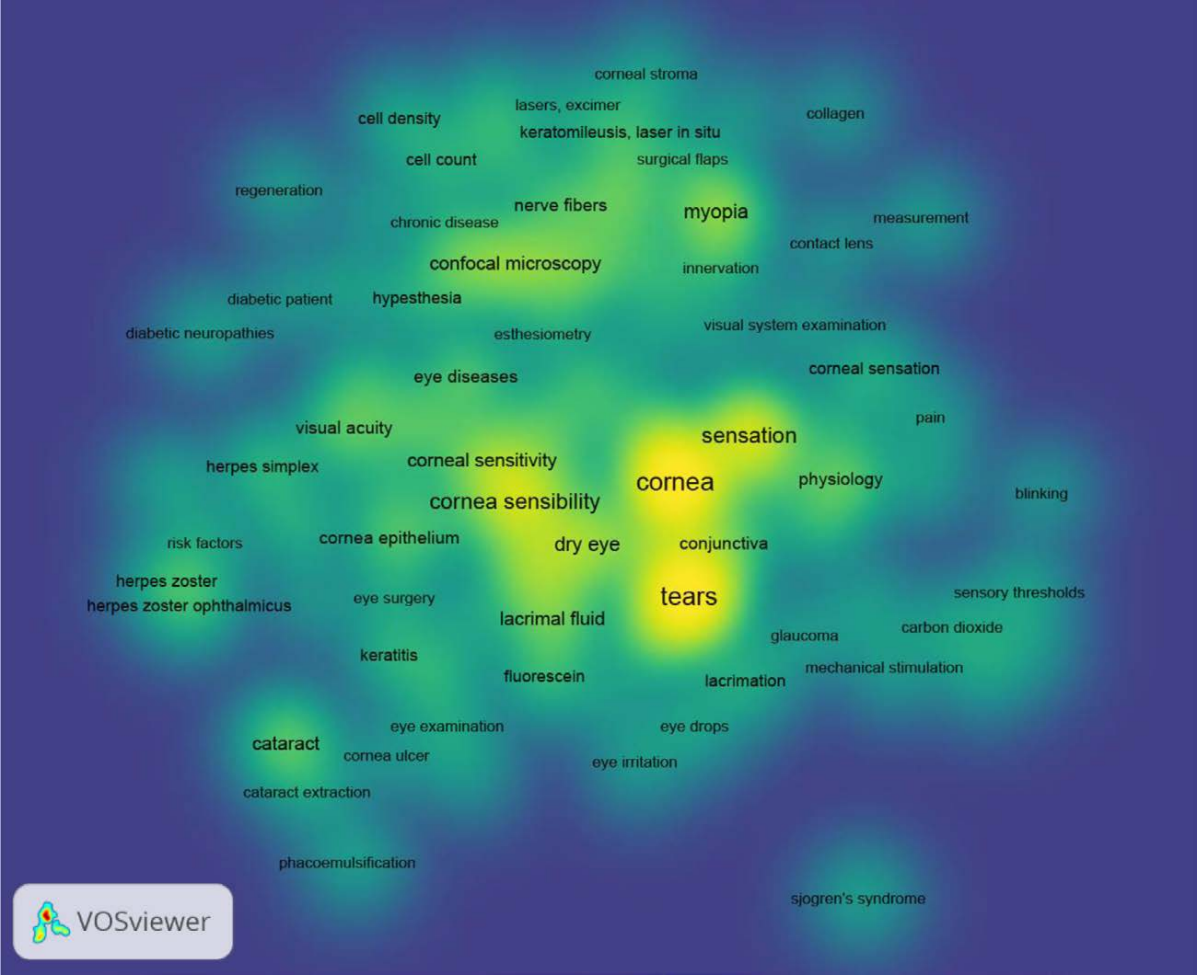
Knowledge mapping network visualization. Density visualization map.

The co-occurrence analysis of authors and keywords identified 55 authors with at least 4 published articles, 15 clusters, and 121 links. With a minimum threshold of 5 occurrences, the keywords formed 6 clusters and 1832 links (see Figs. 6–9).

## 4. Discussion

This work consisted of a bibliometric analysis and mapping knowledge domain of corneal esthesiometry between 2000 and 2024. Bibliometric analysis is a valuable tool for understanding scientific activity in a field of research to determine the production of researchers and their connections, the scientific activity of a country, and how the interest in science in a given field fluctuates over time. In this way, the different branches of health sciences can benefit from efficiently processing multidisciplinary knowledge and capturing the latest development trends to direct future research.^[9]^ Several bibliometric studies on different topics can be found in the field of ophthalmology.^[17–23]^

Two databases were used to cover all possible articles, considering that WoS and Scopus contain the most published medical articles.^[24]^ No languages restrictions were applied, as numerous software tools are now available to facilitate translation. Additionally, no further filters were set, as it was considered that there might be a mismatch between the 2 databases.

Several authors have used the so-called “*h*-index” on different occasions,^[25,26]^ which is a valuable way of measuring how prolific an author or journal is, although it has the limitation of being influenced by the age of the publication.^[27–29]^ For this reason, Figure 4 shows not only the 25 authors with the highest *h*-indexes but also the number of publications for each of them. Figure 4 also shows that Trefford L Simpson was the author with the highest number of published papers; however, it was a medical doctor, not an ophthalmology specialist – Andrew JM Boulton – who had the greatest impact, despite contributing only one-third of the published papers. Of the 25 most prolific authors, 16 were medical doctors, 8 were optometrists and 1 was a biologist, all of whom were affiliated with a university. Forty percent were women.

The 20 journals that published the most on esthesiometry can be found in Table 1. All the included journals belonged to the ophthalmology category, except for the Journal of Refractive Surgery and the Journal of Cataract and Refractive Surgery, which also belong to the surgery category, and PloS One, which belongs to multidisciplinary sciences. Of these, by quartile, in WoS, 10/20 (50%) were Q1, 5/20 (25%) were Q2, 4/20 (20%) were Q3, and 1/20 (5%) were Q4. In Scopus, 15/20 (75%) were Q1, 4/20 (20%) were Q2, and 1/20 (5%) was Q4.

A total of 44 countries were identified as having published research on esthesiometry among the selected articles. The ranking was led by the USA with 81 articles, followed by China with 50 articles. However, the most prolific institution was the Centre for Contact Lens Research School of Optometry at the University of Waterloo in Canada, followed by the University of Manchester in the UK, the Queensland University of Technology in Australia, and the Universidad Miguel Hernández in Spain. More detailed data can be found in Table 3.

**Table 3:** Ranking of the 20 institutions and their countries with the most published papers on esthesiometry.

Articles	Country	Institution
25	Canada	University of Waterloo*
22	United Kingdom	University of Manchester†
19	Australia	Queensland University of Technology
16	Australia	University of New South Wales‡
14	Spain	Universidad Miguel Hernández§
13	United States	University of Miami‖
12	Japan	Keio University
11	United States	Harvard Medical School
9	France	Quinze-Vingts National Ophthalmology Hospital
7	United States	Tufts University School of Medicine
7	United States	Cullen Eye Institute¶
7	Switzerland	University of Applied Sciences and Arts Northwestern Switzerland#
7	South Korea	Institute of Vision Research
7	South Korea	Yonsei University
7	Italy	University of Bologna
7	Spain	University of Valladolid**
6	Turkey	Necmettin Erbakan University
6	New Zealand	University of Auckland
6	United States	Mayo Clinic
6	Canada	University of Toronto

* Includes the Centre for Contact Lens Research School of Optometry.

† Includes the Division of Cardiovascular Medicine, the Faculties of Life Sciences, Medical and Human Sciences and the Institute of Human Development.

‡ Includes the School of Optometry and Vision Science.

§ Includes the Institute of Neurosciences and the Ophthalmologic Institute.

‖ Includes the Bascom Palmer Eye Institute.

¶ Includes the Department of Ophthalmology, Cullen Eye Institute, Baylor College of Medicine.

# Includes the Institute of Optometry.

** Includes the Institute of Applied Ophthalmobiology.

Tools for bibliometric mapping can use 2 types of maps. In a distance-based map, the distance between items reflects the strength of the relationship between them; the greater the distance is, the weaker the strength of the relationship is, in which the strength of the relationship is given by lines connecting the different items. In our work, VOSviewer was used. The size of the label and the circle of an article are determined by the weight of the author or keyword.

The color of an item is determined by the cluster to which it belongs. The lines between elements represent links, and the distance between items in the cluster roughly indicates the relationship of the journals in terms of co-citation links.^[30,31]^ Figure 6 shows the 55 authors who published at least 4 articles, forming 15 clusters of associations. This analysis provides information about which groups may work in the same line of research or perform collaborations. Figure 7 shows the network map of the 92 keywords unified by 6 topics, focusing on corneal pathology (red), structural nerve assessment (yellow), physiology (blue), diabetes (light green), dry eye-Sjögren syndrome (green), and myopia-keratomileusis (purple). The clusters give us an idea of important topics in the domain of esthesiometry and how they relate to other keywords. Another interesting map is the overlay visualization map (Fig. 8), where the same keywords and their connections from the co-occurrence map (Fig. 7) are shown according to the year of publication and may serve to set the trend in terms of clinical utility and the future of further research. Trends in esthesiometry have progressed from refractive surgery (keratomileusis) in the early 2000s – reflecting consolidated studies or studies that have already been extensively explored –, to various pathologies, such as neurotrophic keratitis, diabetic neuropathies, or dry eye disease, in recent years, indicating new lines of research under development or renewed interest. Finally, the density visualization map revealed the keywords with the most occurrences (Fig. 9). Thus, we observed that the terms *cornea*, *sensation*, *tears* and *dry eye* were the most frequent, and were therefore the most influential items in the literature analyzed.

In this study, only WoS and Scopus databases were used as bibliographic sources, given their status as established standards for bibliometric analyses, particularly in the health sciences. Both databases are known for their high-quality metadata, rigorous content selection policies, and robust analytical tools, ensuring methodological validity and reproducibility. While databases such as Dimensions and Crossref have more coverage in subjects such as Social Sciences or Arts and Humanities, and offer broader coverage, they tend to prioritize comprehensiveness over selectivity, which many introduce content of lower scientific relevance or weaker peer-review standards^[32]^ – a critical concern in specific clinical domains such as corneal esthesiometry. Additionally, other databases, such as Microsoft Academic is no longer active, and platforms like Lens of Google Scholar have limited metadata standardization and weak duplicate control, making them less suitable for large-scale, systematic analyses.^[24]^ Scielo was also excluded because of geographic distribution or language limitations. All of the above can be considered a limitation of the present study. We also excluded conferences proceedings and books, which – although they may contain highly valuable contributions – are not peer-reviewed. Another limitation was the time restriction, as only articles published within the last 25 years were included.

In conclusion, this study showed that there is a new and growing trend in the study of esthesiometry, with more articles published in the last 6 months than the average for the previous 25 years. The results revealed that the most central concepts in the literature on corneal sensitivity cluster around cornea, pathophysiology and dry eye, indicating a strong interrelationship between physiological and clinical aspects of the ocular surface. The presence of well-defined thematic clusters suggests that the field is structured around 3 main axes: sensory physiology, ocular pathologies (including infections and surgery), and the mechanism of tear film production and stability. Moreover, the temporal analysis helped identify emerging research trends, with growing interest in topics such as diabetic neuropathies, and dry eye disease-Sjögren syndrome. This reflects a shift toward the study of systemic diseases with ocular involvement and points to new opportunities for developing diagnostic biomarkers related to corneal sensitivity.

## Author contributions

**Conceptualization:** Javier Lozano-Sanroma, Rosa Alvarado-Villacorta.

**Formal analysis:** Javier Lozano-Sanroma, Alberto Barros, Juan Queiruga-Piñeiro, Rosa Alvarado-Villacorta.

**Funding acquisition:** Luis Fernández-Vega Cueto-Felgueroso, Jesús Merayo-Lloves.

**Methodology:** Javier Lozano-Sanroma, Alberto Barros, Juan Queiruga-Piñeiro, Rosa Alvarado-Villacorta.

**Project administration:** Jesús Merayo-Lloves.

**Software:** Javier Lozano-Sanroma, Alberto Barros, Juan Queiruga-Piñeiro.

**Supervision:** Luis Fernández-Vega Cueto-Felgueroso, Jesús Merayo-Lloves.

**Writing – original draft:** Javier Lozano-Sanroma.

**Writing – review & editing:** Ignacio Alcalde.
